# Recapitulation of SARS-CoV-2 infection and cholangiocyte damage with human liver ductal organoids

**DOI:** 10.1007/s13238-020-00718-6

**Published:** 2020-04-17

**Authors:** Bing Zhao, Chao Ni, Ran Gao, Yuyan Wang, Li Yang, Jinsong Wei, Ting Lv, Jianqing Liang, Qisheng Zhang, Wei Xu, Youhua Xie, Xiaoyue Wang, Zhenghong Yuan, Junbo Liang, Rong Zhang, Xinhua Lin

**Affiliations:** 1 State Key Laboratory of Genetic Engineering, School of Life Sciences, Zhongshan Hospital, Fudan University, Shanghai, 200438 China; 2 State Key Laboratory of Medical Molecular Biology, Institute of Basic Medical Sciences, Peking Union Medical College, Chinese Academy of Medical Sciences, Beijing, 100005 China; 3 Key Laboratory of Medical Molecular Virology (MOE/NHC/CAMS), School of Basic Medical Sciences, Shanghai Medical College, Fudan University, Shanghai, 200032 China; 4 Institute of Antibiotics, Huashan Hospital, Fudan University, Shanghai, 200040 China; 5 Sino Organoid Lifesciences Ltd., Shanghai, 201900 China

**Dear Editor,**

The emerging pandemic of coronavirus, SARS-CoV-2 (previously named 2019-nCoV), has posed significant threats to global public health (Wu et al., 2020). The dominant symptoms of coronavirus disease 2019 (COVID-19) are fever and cough (Chen et al., 2020; Huang et al., 2020). However, a proportion of patients showed multi-organ damage and dysfunction (Chen et al., 2020; Huang et al., 2020; Zhu et al., 2020). Of note, liver damage is emerging as a co-existed symptom reported in patients with COVID-19. A recent epidemiologic study in Shanghai (China) reported that 75 out of 148 (50.7%) COVID-19 patients had liver function abnormality, indicated by key liver function parameters above the normal range, including alanine aminotransferase (ALT), aspartate aminotransferase (AST), alkaline phosphatase (ALP) or total bilirubin (TBIL) (Fan et al., 2020). A nation-wide clinical study collecting 1,099 COVID-19 patients revealed that around 20% of patients had elevated ALT and AST and around 10% of patients had elevated TBIL. Especially, the percentage of patients with liver damage is much higher among severe COVID-19 patients than patients with mild symptoms (Huang et al., 2020). Although clinical correlation has been implicated, it remains unclear whether the liver damage is caused by direct virus infection in the liver or by systematic dysfunction such as cytokine storm.

Viruses bind to host receptors to initiate infection. Recent studies have demonstrated that SARS-CoV-2 uses the SARS-CoV receptor angiotensin I converting enzyme 2 (ACE2) for host cell entering and transmembrane serine protease 2 (TMPRSS2) for viral spike (S) protein priming (Kuhn et al., 2004; Hoffmann et al., 2020; Wan et al., 2020; Zhu et al., 2020). It has been shown that ACE2 expression is widely distributed across human tissues, including lung, liver, kidney and multiple digestive tract organs (Qi et al., 2020; Zhang et al., 2020; Zhao et al., 2020). Significant enrichment of ACE2+ population in cholangiocytes compared to hepatocytes in the human healthy liver was reported recently (Chai et al., 2020), implying that SARS-CoV-2 might directly target ACE2+ cholangiocytes in patients. However, whether the virus indeed infects human cholangiocytes thus causes local damage has not been addressed yet.

At present, due to the lack of suitable research models, the mode of virus transmission and tissue tropism is not well established yet. Studies on mechanisms of SARS-CoV-2 pathogenesis mainly depend on bioinformatics analysis, clinical characteristics and rare autopsy reports (Xu et al., 2020). Here we report the use of human organoids as a tool to investigate the SARS-CoV-2 infection and virus-induced tissue damage *ex vivo* at the cellular and molecular levels.

In a three-dimensional (3D) culture system with defined culture medium, liver bile duct-derived progenitor cells embedded in Matrigel can self-assemble into long-term expandable 3D structures that termed “liver ductal organoids”, which retain their tissue-of-origin commitment and genetic stability during self-renewing (Huch et al., 2015). To establish the SARS-CoV-2 infection model with human liver ductal organoids, we first determined whether the long-term organoid culture could preserve the cholangiocytes expressing *ACE2* and *TMPRSS2 ex vivo*. We processed single-cell RNA sequencing (scRNA-seq) to interrogate the transcriptome of cholangiocytes in human liver ductal organoids. A total number of 7,978 cells were analyzed and cell populations were visualized by t-distributed stochastic neighbor embedding (t-SNE), partitioning the cells into 7 clusters (Fig. 1A). The common cholangiocyte markers epithelial cell adhesion molecule (*EPCAM*) and keratin 19 (*KRT19*) were uniformly highly expressed in all the 7 clusters, indicating the heterogeneity of cholangiocytes in these organoids was relatively low (Fig. 1B). Notably, we identified the SARS-CoV-2 receptor gene *ACE2* expressed sparsely among cluster 0–5 in unbiased preferences and was detectable in 2.92% cells (233 out of 7,978) (Fig. 1C and 1D). Anti-ACE2 immunostaining further verified the presence of ACE2+ cholangiocytes in human liver ductal organoids (Fig. 1E). Besides, *TMPRSS2* expressed uniformly across all the clusters and accounted for 51.45% of the total cells (4,105 out of 7,978), it is worth mentioning that 68.24% of the ACE2+ cells were co-expressing *TMPRSS2* (159 out of 233) (Fig. 1C and 1D), making this cell population potentially highly vulnerable to SARS-CoV-2 infection. Interestingly, we found that the cholangiocytes in mouse primary liver ductal organoids had comparable *Epcam* expression but no *Ace2* (mouse *Ace2*) expression (Fig. 1F). Taken together, our data demonstrate that long-term human liver ductal organoid culture preserves the human-specific ACE2+/TMPRSS2+ population of cholangiocytes.

**Figure 1:**
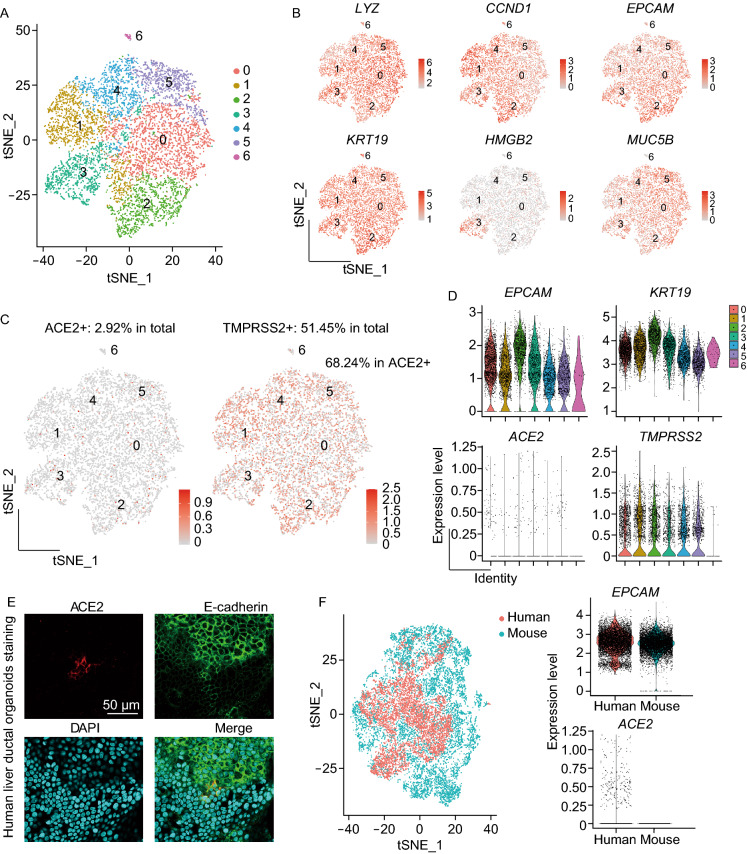
**ACE2+ cholangiocytes are preserved in human liver ductal organoid cultures**. (A) Cell-type clusters. t-SNE visualization of the cell populations (color-coded for clusters) from human liver ductal organoids. (B) t-SNE plots indicating the expression of representative marker genes. (C) t-SNE plots indicating the expression of *ACE2* and *TMPRSS2*. (D) Violin plots showing the expression of representative marker genes. (E) Immunofluorescence staining for ACE2 and E-cadherin in human liver ductal organoids. (F) t-SNE visualization of single cells isolated from human and mouse liver ductal organoids; Violin plots showing the expression of *EPCAM* and *ACE2*

Next, we examined the susceptibility of human liver ductal organoids to SARS-CoV-2. We isolated and plaque-purified the SARS-CoV-2 from a COVID-19 patient in Shanghai. The liver ductal organoids from two individuals were inoculated with SARS-CoV-2 for 1 h then re-embedded in Matrigel and maintained in the culture medium. We performed immunostaining to identify the virus-positive cholangiocytes 24 h post-infection. The expression of SARS-CoV-2 nucleocapsid (N) protein was readily detected in patchy areas of human liver ductal organoids whereas no signal was found in uninfected control (Fig. 2A). In addition, the infected cholangiocytes underwent membrane fusion and formed syncytia (Fig. 2A, enlarge). Although the number of infected cholangiocytes was limited, the examination of the SARS-CoV-2 genomic RNAs revealed a dramatic increase of viral load in organoids at 24 h post-infection (qRT-PCR in Fig. 2B and ORF1 reads count analysis in Fig. 2C). These data demonstrate that human liver ductal organoids are susceptible to SARS-CoV-2 and support robust viral replication. The recapitulation of SARS-CoV-2 infection in human organoids suggests that this model could be employed to dissect the viral pathogenesis and to test antivirals.

**Figure 2:**
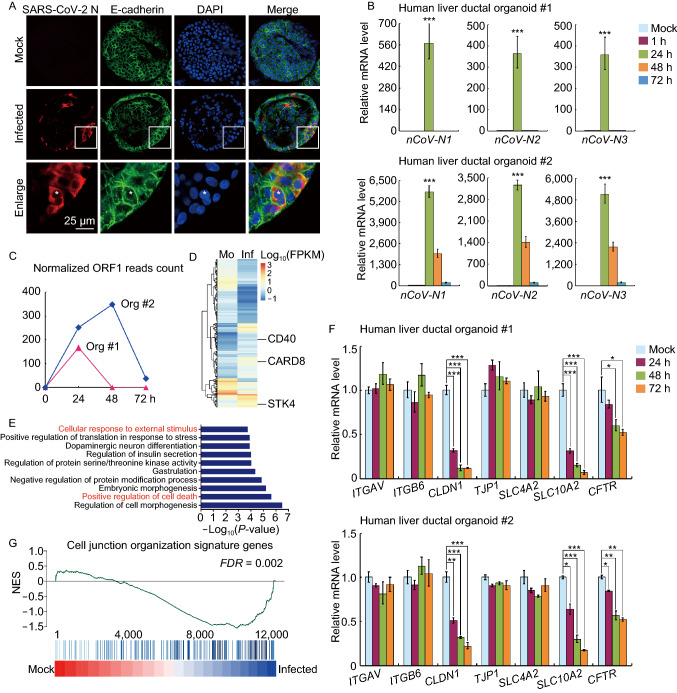
**Recapitulation of SARS-CoV-2 infection in human liver ductal organoids**. (A) Immunofluorescence staining for SARS-CoV-2 N protein and E-cadherin in human liver ductal organoids. (B) Two cases of human liver ductal organoids were harvested at indicated time points following SARS-CoV-2 infection to examine the virus load using qRT-PCR. *RNP* was used as an internal control. (C) Changes in expression of ORF1 in human liver ductal organoids (Org #1 and Org #2) in response to SARS-CoV-2 infection at indicated times. (D) Heatmap showing differentially expressed genes (DEGs) in SARS-CoV-2-infected organoids (Inf) (24 h) versus the mock (Mo). (E) GO analysis of DEGs. Top 10 enriched biological processes were shown. (F) Two cases of human liver ductal organoids after SARS-CoV-2 infection were harvested to examine the expression of indicated genes using qRT-PCR. *GAPDH* was used as an internal control. Data were presented as mean ± s.d. * indicates *P* < 0.05; ** indicates *P* < 0.01; *** indicates *P* < 0.001 (G) GSEA enrichment analysis of SARS-CoV-2-infected organoids (48 h) versus the mock for cell junction organization signature genes

The viral load in organoids was significantly decreased at 48 h post-infection (Fig. 2B), probably due to virus-induced death of host cholangiocytes or activation of anti-viral response. Gene transcriptome examination by RNA-sequencing revealed a set of 337 differentially expressed genes (DEGs) in SARS-CoV-2-infected organoids (Fig. 2D, *P* < 0.01). Gene ontology (GO) analysis highlighted intensive expression alteration of genes involved in “positive regulation of cell death” and “cellular response to external stimulus” (Fig. 2E). Consistently, SARS-CoV-2 infection stimulated the expression of several critical cell apoptosis factors, represented by CD40 molecule (*CD40*), caspase recruitment domain family member 8 (*CARD8*) and serine/threonine kinase 4 (*STK4*) (Fig. 2D). These data indicate that SARS-CoV-2 infection induces cell death of host cholangiocytes.

We then testified whether SARS-CoV-2 infection and consequent cell death could influence the tissue behavior. The main function of cholangiocytes in homeostasis is to transport bile acid secreted by hepatocytes into bile ducts. The tight junction between cholangiocytes maintains the barrier function of bile ductal epithelium, which is essential for bile acid collection and excretion. We found that SARS-CoV-2 infection ablated the expression of claudin 1 (*CLDN1*) (Fig. 2F). In addition, gene set enrichment analysis (GSEA) indicated that SARS-CoV-2-infected organoids had decreased enrichment of cell junction organization signature genes (Fig. 2G), suggesting that the barrier function of bile ductal epithelium was disrupted. More importantly, the expression of two major bile acid transporter genes, solute carrier family 10 member 2 (*SLC10A2*) and cystic fibrosis transmembrane conductance regulator (*CFTR*), was significantly decreased following SARS-CoV-2 infection (Fig. 2F). These data indicate that SARS-CoV-2 infection impairs the barrier and bile acid transporting functions of cholangiocytes through modulating the expression of genes involved in tight junction formation and bile acid transportation. Our study therefore supports the idea that the liver damage in COVID-19 patients might result from direct cholangiocyte injury and consequent bile acid accumulation induced by viral infection.

Organoids retain the biology of individual tissues, which hold great promise for the study of host-microbe interaction (Dutta and Clevers, 2017). Here, by single-cell RNA sequencing, we demonstrated that long-term liver ductal organoid culture preserves the human-specific ACE2+ population of cholangiocytes. Moreover, human liver ductal organoids were permissive to SARS-CoV-2 infection and support robust replication. To our knowledge, this is the first SARS-CoV-2-human organoid infection model reported. Given that the culture conditions for various organoids (lung, intestine, and kidney) have already been established, it would be intriguing to study the tropism, replication, and innate immune response of SARS-CoV-2 infection in other organs that are targeted by this virus.

Liver damage is a common feature in severe COVID-19 patients. The improper use of anti-viral drugs may cause hepatotoxicity thus liver damage. Besides, SARS-CoV-2 infection may trigger an overwhelming inflammatory response, which leads to multi-organ injuries (Huang et al., 2020). In this study, we found that virus infection impairs the barrier and bile acid transporting functions of cholangiocytes through the dysregulation of genes involved in tight junction formation and bile acid transportation. This could be due to the direct viral cytopathogenic effect on target cells that express *ACE2* and *TMPRSS2*. Therefore, it is of importance to consider that the liver damage in COVID-19 patients might be in part the result of direct cholangiocyte injury and consequent bile acid accumulation caused by SARS-CoV-2 infection.

By employing human liver ductal organoids, we investigated the infection and liver tissue damage of SARS-CoV-2 *ex vivo*. These results indicate that control of liver damage caused directly by viral infection should be valued in treating COVID-19 patients. Our findings also provide an application of human organoids in investigating the tropism and pathogenesis of SARS-CoV-2, which would facilitate novel drug discovery.

## Footnotes

We thank Dr. Stacey S. Huppert and Dr. Xiaofei Yu for technical support and discussion. We also wish to acknowledge Di Qu, Xia Cai, Zhiping Sun, Wendong Han and the others at Biosafety Level 3 Laboratory of Fudan University for experiment design and expert technical assistance. This work was supported by grants from the National Key Research and Development Program of China (2018YFA0109400 and 2018YFA0109800), the National Natural Science Foundation of China (31730044 and 32041005), the Zhejiang University Special Scientific Research Fund for COVID-19 Prevention and Control (2020XGZX013) and the Shanghai Municipal Science and Technology Major Project (2017SHZDZX01).

The authors declare that they have no conflict of interest.

All procedures followed were in accordance with the ethical standards of the Medical Ethical Council of Zhongshan Hospital and with the Helsinki Declaration of 1975, as revised in 2000 (5). Informed consent was obtained from all patients for being included in the study.

B.Z., C.N. and R.Z. conceived the study; B.Z., C.N., R.G., Y.W., L.Y., J.W., T.L., J.L., Q.Z., W.X. and R.Z. performed the experiments; B.Z., J.L., R.Z. and X.L. supervised the work; Y.X., X.W. and Z.Y. contributed to the discussion of the results; and B.Z., C.N., R.Z. and X.L. wrote the manuscript.

## Electronic supplementary material

Below is the link to the electronic supplementary material.

Supplementary material 1 (PDF 380 kb)
