# Supplementary material for: Recapitulation of SARS-CoV-2 infection and cholangiocyte damage with human liver ductal organoids
Source: Protein Cell. 2020 Apr 17;11(10):771–5. doi: 10.1007/s13238-020-00718-6 (PMC7164704; doi:10.1007/s13238-020-00718-6)
Supplement: 13238_2020_718_MOESM1_ESM — Supplementary Information [file 13238_2020_718_moesm1_esm.pdf]

Supplementary Information

Recapitulation of SARS-CoV-2 Infection and Cholangiocyte Damage with Human Liver Ductal Organoids

Zhao et al.

Methods

Supplementary Table 1-2

Methods

Human biopsy. Human liver biopsies were obtained and used for research purposes with approval from the Medical Ethical Council of Zhongshan Hospital. The study abides by the Declaration of Helsinki principles.

Virus stock preparation. SARS-CoV-2 was isolated from a COVID-19 patient in Shanghai, China (SARS-CoV-2/SH01/human/2020/CHN, GenBank accession no. MT121215). Virus was plaque-purified, propagated in Vero-E6 cells, and stored at –80°C for use. All experiments involving virus infections were done in biosafety level 3 facility strictly following the regulations.

Liver ductal organoid culture and SARS-CoV-2 infection. The human ductal organoids were generated from primary bile ducts isolated from human liver biopsies. The organoids embedded in Matrigel (BD Bioscience, 354230) were scrambled off the plate and collected in tubes, then washed with cold PBS by pipetting the material up and down for 10 times. After centrifugation (2 min at 250 g), the organoid pellet was suspended with medium containing 5 μM Y-27632 (Sigma-Aldrich, Y0503). Around 200-300 organoids were infected with 1.2×10^5 PFU of SARS-CoV-2 in 24 well plate containing 500uL medium and incubated at 37 °C for 1 hour. After incubation, organoids were

collected by pipetting and washed once with PBS, then repeated the centrifugation and removed supernatant. The organoids were embedded with Matrigel, followed by seeding on a 24-well plate. After polymerization, culture medium was added.

Immunofluorescence. For whole mounting liver organoids staining, organoids were fixed in 4% paraformaldehyde for 30 min at 4 °C, washed with PBS and permeabilized with 0.25% Triton X-100 (Sigma-Aldrich, X100) in PBS for 30 min. The organoids were then washed with PBST (PBS containing 0.1% Tween 20) and blocked by 5% BSA in PBST for 1 hour at room temperature. Organoids were incubated with the primary antibodies at 4 °C overnight, washed with PBST 3 times, and incubated with the secondary antibodies and DAPI for 1 hour at room temperature. Organoids imaging was performed on confocal microscope (OLYMPUS, FV3000). The following antibodies were used: rabbit anti-ACE2 (Sino Biological Inc, 10108-RP01, 1:100), rabbit anti-SARS-CoV-2 N protein (Rockland, 200-401-A50, 1:500), mouse anti-E-cadherin (BD Biosciences, 610181), Cy3-conjugated donkey anti-rabbit IgG (Jackson Lab,711-165-152), Alexa Fluor 488-conjugated donkey anti-mouse IgG (Jackson Lab, 715-545-151).

Quantitative RT-PCR. Total RNA was isolated from organoids by RNeasy Mini kit (QIAGEN,74106) and reverse-transcribed into cDNA with M-MLV Reverse Transcriptase (Invitrogen, 28025013) in biosafety level 2 facility strictly following the regulations.

Quantitative real-time PCR was performed on CFX384 Touch System (Bio Rad). Primers used were listed in Supplementary Table 2. The SARS-CoV-2 primer and probe sets were provided by Integrated DNA Technologies (IDT, 10006606).

Single-cell RNA seq and data analysis. Single-cell RNA sequencing was performed using the 10x Genomics Chromium System. Human liver ductal organoids were derived from a patient who underwent resection, cultured for 3 passages as described above. Mouse primary liver ductal organoids were cultured from biliary ducts isolated from an 8-week-old C57BL/6 mouse. Briefly, organoids were dissociated with 1× TrypLE Select Enzyme (Gibco, 12563011) to obtain single cell suspension. A total of around 8,000 cells per sample were captured on a 10×Chromium device and library preparation was carried out using Single Cell 3’ Reagent Kits v2 according to the manufacturer’s instructions (10× Genomics). Libraries were sequenced on an Illumina NovaSeq 6000 platform.

Cell Ranger (version 3.1) with default parameters was used to process sequencing data to generate feature-barcode matrices. The human dataset was analyzed using the standard workflow on the Seurat R Package (version 3.1.3). For the feature-barcode matrix of 8,094 cells from the human dataset, we removed cells with less than 500 genes and more than 6,000 genes as well as cells with high fraction of mitochondrial UMIs (> 20%). 7,978 high quality cells and 17,447 expressed genes were remained for downstream analysis. The cell populations were clustered using the ‘FindClusters’ function and

visualized in 2 dimensions by t-distributed stochastic neighbor embedding (t-SNE) derived from the top 10 principal components. For the feature-barcode matrix of 9,690 cells from the mouse dataset, we retained cells with expressed genes between 500 and 6,000 as well as cells with low fraction of mitochondrial UMIs (< 10%). Finally, 8,812 high quality cells and 16,019 expressed genes were remained for downstream analysis. The integration of human and mouse datasets was processed by the standard Seurat v3 integration workflow.

Bulk RNA-seq and analysis. Total RNA was extracted from liver organoids by RNeasy Mini Kit (QIAGEN) following manufacturer's instructions and processed with the Ovation® RNA-Seq System V2 Kit (NuGEN) to produce libraries for deep sequencing. Total RNA extraction and library preparation were performed in biosafety level 3 facility according to strict regulations. Libraries were sequenced on an Illumina NovaSeq 6000 platform. After quality control, clean reads were aligned to human reference genome (GRCh38) using HISAT2 (version 2.1.0). The alignments were then passed to StringTie (version 1.3.5) to assemble and quantify the transcripts in each sample. Differentially expressed genes (DEGs) was identified by the R package edgeR (version 3.28.1). Genes were defined as DEGs if it possesses the following characteristics: 1) gene expression (FPKM) > 1 in any sample, 2) absolute log2 (fold change) > 2 and 3) *p*-value < 0.01. Visualization and hierarchical clustering of log2-transformed FPKM was generated by pheatmap (version

1.0.12). GO analysis was performed using metascape (http://metascape.org). Gene set enrichment analysis was performed with GSEA v3.0 software (available from the Broad Institute).

Statistical analysis. We employed Student's *t*-test or ANOVA test to analyze the parametric experimental results. Significant differences were noted with asterisks.

Supplementary Table 1. 337 differentially expressed genes (DEGs) in SARS-CoV-2-infected organoids (24 h) versus the mock.

Gene Symbol	log2FoldChange	*P*-value
BLOC1S5-TXNDC5	-17.93	1.36E-22
LPIN3	10.57	3.33E-17
CARMIL1	-10.19	9.25E-17
CDC42EP4	-10.40	4.50E-16
USP2	-10.11	1.55E-15
PPP1CB	-9.11	9.07E-15
SENP3-EIF4A1	-13.55	2.27E-14
AC011511.4	9.88	3.08E-14
ZNF662	8.76	1.24E-13
DEDD2	-8.90	5.48E-13
DAG1	-8.17	9.07E-13
XYLT2	8.00	1.22E-12
ATXN7L3	-8.22	2.04E-12
DDAH1	7.84	2.69E-12
BROX	-7.81	2.73E-12
UBE2D4	-7.61	7.05E-12
CCL15-CCL14	-12.14	9.45E-12
TMEM263	7.50	1.05E-11
ARC	-12.11	1.11E-11
FOXA2	-7.86	1.19E-11
PIK3C2B	7.44	1.48E-11
SRGAP1	7.35	1.87E-11
CNP	-7.33	1.91E-11
GPR75-ASB3	8.37	1.97E-11
TCTN2	-7.21	5.17E-11
FBXO34	-7.38	5.25E-11
C17orf99	-11.69	6.43E-11
ZNF282	-7.29	7.58E-11
AC008763.3	11.57	1.10E-10
TULP2	-11.54	1.25E-10
TXLNA	-6.89	1.30E-10
KRTAP4-1	-11.38	2.45E-10
PCMTD2	-6.75	2.70E-10
ASGR2	11.30	3.54E-10
ST6GAL2	6.69	4.30E-10
TMEM144	-6.56	5.72E-10

RIN2	-6.51	6.75E-10
GAS2	-6.54	8.72E-10
CEMP1	-11.00	1.20E-09
IL6R	-6.68	1.22E-09
MGAT1	6.28	2.11E-09
CDKAL1	6.23	2.33E-09
C10orf113	-6.87	3.15E-09
TXNDC5	6.14	3.21E-09
EPPK1	-6.15	3.49E-09
LZTS3	-6.28	3.49E-09
DPYSL2	-6.04	4.62E-09
HNRNPH2	-6.06	4.90E-09
AC012309.1	-6.38	6.42E-09
FOSB	-5.93	8.36E-09
VDR	-5.83	1.37E-08
ROBO1	5.79	1.75E-08
MAFF	5.75	2.01E-08
HAUS5	-5.67	2.71E-08
MACC1	-5.63	2.73E-08
NEU3	5.60	4.56E-08
MED22	5.64	7.02E-08
PRPS2	5.31	1.02E-07
IBA57	-5.18	1.71E-07
EFNA5	5.18	1.73E-07
ELOVL7	5.30	2.18E-07
ZC3H11A	-5.14	2.26E-07
CDC42SE2	5.09	2.56E-07
OVCA2	5.12	3.24E-07
ZDHHC20	5.02	4.01E-07
ADM	-5.08	4.09E-07
PDP2	4.98	4.29E-07
ZBTB43	-4.96	4.48E-07
ZNF331	5.08	4.51E-07
CCNF	-4.94	4.56E-07
SLC17A9	-5.22	4.57E-07
ZNF445	4.94	5.41E-07
CNEP1R1	-4.80	8.93E-07
TNRC6C	-4.81	1.05E-06
TRIM72	-4.78	1.10E-06

IL3RA	-4.99	1.14E-06
AKAP2	4.72	1.44E-06
FARP2	-4.66	1.46E-06
FANCD2	4.63	1.61E-06
EPG5	-4.64	1.74E-06
C7orf25	4.62	1.97E-06
AC023055.1	-4.66	2.13E-06
AP001931.1	-5.49	2.22E-06
ZNF564	-4.76	2.29E-06
TUBB3	4.54	2.51E-06
TACC1	-4.48	2.98E-06
C6orf201	-9.04	3.11E-06
MEGF8	4.48	3.22E-06
DGKQ	-4.51	3.24E-06
LCLAT1	4.45	3.52E-06
CCDC113	4.44	3.75E-06
BTBD9	4.38	4.42E-06
ZNF672	-4.47	4.42E-06
GLI2	4.39	5.43E-06
SLC35E2B	4.33	6.03E-06
DDX3X	-4.30	6.08E-06
FAM111A	4.28	6.71E-06
ITPK1	4.26	7.59E-06
RPL36A-HNRNPH2	4.26	8.17E-06
YEATS2	-4.26	8.53E-06
BTBD11	4.22	9.51E-06
HNF4A	-4.18	9.69E-06
EDEM3	-4.18	1.16E-05
TAF7	4.13	1.24E-05
SERPINB2	4.12	1.26E-05
MFAP2	-4.40	1.66E-05
APOBEC3D	-4.19	1.81E-05
ZBTB8A	4.06	1.81E-05
C10orf10	-4.22	1.84E-05
RC3H1	-3.98	2.34E-05
RRAGD	-4.00	2.44E-05
HIPK3	-3.99	2.50E-05
ADH1A	4.17	2.60E-05
XRRA1	3.92	2.72E-05

SLC10A1	-4.13	2.80E-05
PFAS	-3.88	3.15E-05
ZNF485	3.93	3.50E-05
PAQR6	-4.01	3.64E-05
MAK	3.88	3.72E-05
CDK3	4.00	3.84E-05
SVIL	3.82	4.10E-05
RAB3A	3.91	4.45E-05
CTDSP1	3.80	4.50E-05
IL1RL1	3.79	4.53E-05
NAV3	3.82	4.63E-05
WDR37	-3.81	4.71E-05
ACP5	-3.99	4.79E-05
CELA3A	-4.24	5.28E-05
SPRR1B	3.76	5.30E-05
MYLIP	-3.76	5.35E-05
STK4	3.72	5.84E-05
AC012254.2	-4.39	6.56E-05
ORAI2	3.68	6.98E-05
MPZL1	3.67	7.20E-05
FBXO32	-3.67	7.22E-05
DSC2	3.66	7.39E-05
ZFX	3.65	7.74E-05
CPD	-3.65	7.98E-05
AC036214.3	-3.75	8.49E-05
PDS5B	-3.60	9.34E-05
XIAP	3.59	9.78E-05
AP4E1	3.55	1.24E-04
KCTD6	3.61	1.36E-04
ATP6V1C2	-3.52	1.37E-04
CD109	-3.49	1.40E-04
AC004922.1	-3.50	1.42E-04
ZFP90	3.49	1.53E-04
POLDIP3	3.45	1.67E-04
VAMP1	-3.46	1.70E-04
CDKN2AIP	3.43	1.98E-04
CTNNB1	-3.39	1.99E-04
TUBB2B	-3.41	2.08E-04
GATD1	-3.40	2.11E-04

LCA5L	3.45	2.21E-04
ZBTB3	3.38	2.23E-04
FAM210A	3.34	2.44E-04
CYB5D1	3.35	2.53E-04
ZNF559	3.39	2.54E-04
TIAM1	3.33	2.58E-04
PDCD4	-3.32	2.58E-04
FAM214B	3.34	2.70E-04
AL109811.4	-3.41	2.96E-04
ANKRD1	3.28	3.05E-04
CREB3L2	-3.32	3.13E-04
AGFG2	3.26	3.33E-04
ZBTB20	-3.25	3.40E-04
CARD8	3.23	3.71E-04
PEA15	3.22	3.81E-04
NUDT17	-3.35	3.87E-04
B3GNT4	-3.26	3.90E-04
UBE4A	3.21	3.90E-04
SAMD4A	-3.25	3.98E-04
NIPSNAP1	-3.20	4.10E-04
NPIPB15	-3.22	4.24E-04
HFE	3.18	4.47E-04
ZDHHC14	-3.17	5.17E-04
CNNM2	3.14	5.30E-04
ZNF234	3.15	5.46E-04
TAB2	3.13	5.54E-04
EPB41L1	-3.11	5.62E-04
GRB2	-3.11	5.68E-04
NCK2	-3.11	5.96E-04
PYCR3	-3.13	6.06E-04
ASAP2	3.09	6.07E-04
KLF4	-3.08	6.32E-04
ARHGAP26	-3.07	6.47E-04
ATF3	-3.10	6.62E-04
COCH	-3.18	6.67E-04
MRNIP	-3.06	6.73E-04
KIAA1683	-3.22	6.93E-04
AFMID	3.05	7.00E-04
C8orf82	-3.08	7.37E-04

ESAM	-3.12	7.47E-04
RND1	-3.03	7.51E-04
CD40	3.11	8.10E-04
CAB39	3.00	8.46E-04
POU5F1	-3.07	8.83E-04
LRRC75A	-2.98	8.93E-04
CANT1	2.97	9.51E-04
PAPD5	2.96	9.69E-04
YOD1	-2.96	9.76E-04
DAB2IP	2.97	9.92E-04
AC023509.3	-3.08	9.97E-04
FLVCR1	-2.93	1.08E-03
CCDC40	-3.02	1.08E-03
CHP1	-2.91	1.11E-03
C18orf25	-2.90	1.16E-03
TCF7	2.92	1.17E-03
RAB8B	2.92	1.18E-03
ZRSR1	-3.00	1.24E-03
FAM46C	-2.88	1.24E-03
ERC1	-2.90	1.24E-03
COQ10B	-2.87	1.29E-03
STIM1	-2.87	1.34E-03
PLA2G15	2.86	1.36E-03
MLLT6	2.87	1.40E-03
FKBP9	-2.85	1.41E-03
C6orf15	2.95	1.44E-03
PLSCR3	2.85	1.44E-03
AC005324.3	2.84	1.45E-03
LRP2BP	-2.87	1.45E-03
KIAA1217	-2.84	1.47E-03
ZCCHC2	-2.84	1.50E-03
STYX	2.83	1.52E-03
URM1	2.82	1.54E-03
TSKU	2.84	1.55E-03
RNF146	2.82	1.58E-03
MMP10	2.82	1.74E-03
ITGA6	2.78	1.79E-03
HLCS	2.79	1.79E-03
PIP5KL1	-2.87	1.86E-03

WDR89	2.77	1.91E-03
LUZP2	-2.79	1.95E-03
METTL15	2.76	1.95E-03
QKI	-2.73	2.10E-03
SLC44A1	2.72	2.18E-03
RBM12	2.72	2.21E-03
CNTNAP2	2.72	2.22E-03
DUSP2	-2.75	2.29E-03
UBE2Z	-2.69	2.39E-03
CCNG2	-2.71	2.48E-03
PRR14L	2.67	2.59E-03
XAGE1A	-2.87	2.60E-03
ZBTB40	2.67	2.60E-03
HIST2H2AC	-2.81	2.68E-03
ZNF223	2.72	2.71E-03
TMED2	-2.64	2.83E-03
TRIM39	2.65	2.89E-03
LRP5L	-2.66	3.04E-03
ZNF239	2.66	3.05E-03
TMEM250	-2.65	3.08E-03
DHDH	-2.76	3.10E-03
ARMT1	2.61	3.21E-03
EMC6	2.68	3.23E-03
FAM57A	-2.61	3.24E-03
CC2D1B	2.59	3.40E-03
SNX27	-2.58	3.46E-03
DKK1	2.58	3.49E-03
KDM6B	-2.59	3.56E-03
MUC6	-2.56	3.71E-03
SPATA12	-2.60	3.74E-03
TTC21A	-2.63	3.75E-03
HIST1H3A	-2.75	3.87E-03
BTBD19	-2.54	3.92E-03
SPECC1L	-2.56	3.96E-03
PIFO	-2.64	4.00E-03
WSCD1	-2.55	4.04E-03
ANKRD37	-2.57	4.06E-03
ANO1	-2.53	4.10E-03
VSNL1	2.57	4.11E-03

SERPINA6	2.58	4.17E-03
WASF3	2.54	4.24E-03
SPON1	-2.59	4.33E-03
DDT	-2.51	4.39E-03
U2AF2	2.52	4.39E-03
GCNT4	2.50	4.48E-03
SH3D19	-2.50	4.56E-03
TBL1XR1	2.49	4.67E-03
GALNS	-2.50	4.70E-03
C12orf60	2.51	4.77E-03
IRAK1BP1	2.50	4.80E-03
ERO1B	-2.50	4.93E-03
ALDH5A1	-2.47	4.97E-03
ATP8B3	-2.52	5.03E-03
DHRS4L2	2.49	5.03E-03
YWHAB	-2.44	5.31E-03
CLN6	2.45	5.36E-03
PTBP2	-2.44	5.48E-03
DUSP6	2.43	5.49E-03
NCKAP5L	-2.45	5.67E-03
ARHGAP33	-2.54	5.74E-03
MANEAL	2.42	5.88E-03
SAPCD1	-2.49	6.04E-03
USP30	2.44	6.04E-03
PPP3CA	2.40	6.06E-03
NFKBIZ	-2.40	6.13E-03
EBLN2	-2.44	6.18E-03
ZNF816	2.41	6.18E-03
AURKC	-2.49	6.19E-03
RGS14	2.42	6.24E-03
GATAD2A	-2.39	6.35E-03
IRF1	-2.39	6.35E-03
ACSF2	-2.41	6.42E-03
AP002990.1	-2.39	6.43E-03
GAST	2.55	6.45E-03
CCDC137	2.38	6.46E-03
MAP3K8	-2.40	6.58E-03
CCNL1	-2.38	6.62E-03
NPIPB12	-2.42	6.72E-03

ARSJ	2.37	6.81E-03
ARHGDIA	2.37	6.81E-03
POLG2	-2.35	7.28E-03
SLC51A	-2.40	7.34E-03
GABBR1	-2.37	7.39E-03
TLE3	2.34	7.49E-03
APBB2	-2.34	7.54E-03
DFNA5	2.35	7.62E-03
PPP1R15A	-2.33	7.85E-03
CHN1	-2.39	7.97E-03
NPIPB4	-2.33	8.06E-03
CUX1	2.31	8.16E-03
NPEPPS	2.31	8.17E-03
PARS2	2.31	8.39E-03
CISD3	-2.29	8.59E-03
SOCS3	-2.30	8.60E-03
RELB	-2.31	8.61E-03
RTL8C	2.33	8.63E-03
FOS	-2.29	8.69E-03
ZSCAN21	2.30	8.69E-03
TSHZ1	2.30	8.79E-03
ZNF528	2.33	8.96E-03
HLA-DRA	2.29	9.24E-03
XAGE1B	-2.43	9.26E-03
NR4A2	-2.28	9.37E-03
RARB	2.28	9.59E-03
TTLL3	-2.27	9.73E-03
ARF1	2.25	9.91E-03

Supplementary Table 2. Primers and probes for qPCR.

TaqMan primers and probes	Oligonucleotide sequence (5'-3')
nCov-N1 forward	GACCCCAAAATCAGCGAAAT
nCov-N1 reverse	TCTGGTTACTGCCAGTTGAATCTG
nCov-N1 probe	FAM-ACCCCGCATTACGTTTGGTGGACC-BHQ1
nCov-N2 forward	TTACAAACATTGGCCGCAAA
nCov-N2 reverse	GCGCGACATTCCGAAGAA
nCov-N2 probe	FAM-ACAATTTGCCCCCAGCGCTTCAG-BHQ1
nCov-N3 forward	GGGAGCCTTGAATACACCAAAA
nCov-N3 reverse	TGTAGCACGATTGCAGCATTG
nCov-N3 probe	FAM-AYCACATTGGCACCCGCAATCCTG-BHQ1
RNP forward	AGATTTGGACCTGCGAGCG
RNP reverse	GAGCGGCTGTCTCCACAAGT
RNP probe	FAM-TTCTGACCTGAAGGCTCTGCGCG-BHQ1

qPCR primers	Forward (5'-3')	Reverse (5'-3')
GAPDH	GGTATCGTGGAAGGACTCATGAC	ATGCCAGTGAGCTTCCCGTTCAG
ITGAV	GGGATGACAACCCTCTGAC	GTTTCTCAGCTCATAGATGTG
ITGB6	CTGCTTTGCCTGTTCTTTCTATTTC	GTTTCTGCACCTCCCAGGG
CLDN1	GTGCGATATTTCTTCTTGCAGGTC	TTCGTACCTGGCATTGACTGG
TJP1	GTGTTGTGGATACCTTGT	GATGATGCCTCGTTCTAC
SLC4A2	TCCTCCCACCACATCCATCA	CTCCTCAATGGTCGGGGTTTC
SLC10A2	CAGTTTGGAATCATGCCCCTC	GCTATGAGCACAATGAGGATGG

CFTR	TGACCTTCTGCCTCTTACCA	CACTATCACTGGCACTGTTGC
